# Quantitative Proteomics Reveals Ecophysiological Effects of Light and Silver Stress on the Mixotrophic Protist *Poterioochromonas malhamensis*

**DOI:** 10.1371/journal.pone.0168183

**Published:** 2017-01-05

**Authors:** Daniela Beisser, Farnusch Kaschani, Nadine Graupner, Lars Grossmann, Manfred Jensen, Sabrina Ninck, Florian Schulz, Sven Rahmann, Jens Boenigk, Markus Kaiser

**Affiliations:** 1 Genome Informatics, Institute of Human Genetics, University Hospital Essen, University of Duisburg-Essen, Hufelandstr. 55, 45147 Essen, Germany; 2 Biodiversity, Faculty of Biology, University of Duisburg-Essen, Universitätsstr. 5, 45141 Essen, Germany; 3 Chemical Biology, Center for Medical Biotechnology (ZMB), Faculty of Biology, University of Duisburg-Essen, Universitätsstr. 2, 45117 Essen, Germany; 4 Centre for Water and Environmental Research (ZWU), University of Duisburg-Essen, Universitätsstr. 2, 45117 Essen, Germany; Pacific Northwest National Laboratory, UNITED STATES

## Abstract

Aquatic environments are heavily impacted by human activities including climate warming and the introduction of xenobiotics. Due to the application of silver nanoparticles as bactericidal agent the introduction of silver into the environment strongly has increased during the past years. Silver ions affect the primary metabolism of algae, in particular photosynthesis. Mixotrophic algae are an interesting test case as they do not exclusively rely on photosynthesis which may attenuate the harmful effect of silver. In order to study the effect of silver ions on mixotrophs, cultures of the chrysophyte *Poterioochromonas malhamensis* were treated in a replicate design in light and darkness with silver nitrate at a sub-lethal concentration. At five time points samples were taken for the identification and quantitation of proteins by mass spectrometry. In our analysis, relative quantitative protein mass spectrometry has shown to be a useful tool for functional analyses in conjunction with transcriptome reference sequences. A total of 3,952 proteins in 63 samples were identified and quantified, mapping to 4,829 transcripts of the sequenced and assembled transcriptome. Among them, 720 and 104 proteins performing various cellular functions were differentially expressed after eight days in light versus darkness and after three days of silver treatment, respectively. Specifically pathways of the energy and primary carbon metabolism were differentially affected by light and the utilization of expensive reactions hints to an energy surplus of *P. malhamensis* under light conditions. The excess energy is not invested in growth, but in the synthesis of storage metabolites. The effects of silver were less explicit, observable especially in the dark treatments where the light effect could not mask coinciding but weaker effects of silver. Photosynthesis, particularly the light harvesting complexes, and several sulphur containing enzymes were affected presumably due to a direct interference with the silver ions, mainly affecting energy supply.

## Introduction

Protist predators are the primary agents of top-down control of bacteria and primary producers, and thus key organisms determining the fate and transport of organic matter in nature [1]. Shifts in species composition of protist communities as well as changes in their functional ecology will therefore affect the overall architecture of aquatic food webs. Due to the immense diversity of protists and their small size the ecology of these organisms has, up to recently, largely been addressed on the level of broad functional groups.

Protists, as e.g. small flagellates, comprise a large number of taxonomically quite different organisms with different growth and survival strategies ranging from mainly photosynthetic flagellates (e.g. *Uroglena*), that only take up very small numbers of bacteria, probably as vitamin source, to entirely heterotrophic flagellates (e.g. *Spumella* spp.). Between these extremes, there are organisms that utilize both photosynthesis and phagotrophy to different extents depending on the environmental conditions experienced (e.g. [2, 3]). Mixotrophy is particularly widespread in chrysophytes (throughout the manuscript, this term refers to the chrysophytes sensu lato, comprising the Synurales). Further, chrysophytes are of special interest because their abundance may increase during phases of lake re-oligotrophication [4, 5] and because they often dominate mixotrophic phytoplankton populations [6].

Newly developed molecular methods now allow for deeper insights into the ecology of these organisms and with that into the function and the regulation of processes within the microbial food web.

Light and food availability as well as stressors such as heavy metals can have a crucial impact on community structure and functioning [7–9]. Environmental stressors will become more and more important due to ongoing environmental changes. Pollution is among the most serious stressors affecting the composition of aquatic communities and the well-being of organisms [10]. Among metal pollutants silver ions are one of the most toxic forms, and have thus been assigned to the highest toxicity class, together with cadmium, chromium (VI), copper and mercury [11]. Its toxicity to a wide range of microorganisms combined with its low toxicity to humans led to the development of a wealth of silver-based products in many bactericidal applications [12, 13] accounting, for example, for more than 1000 nanotechnology-based consumer products [14]. Accordingly, silver is a widely distributed metal in the environment originating from its different forms of application as metal, salt and nanoparticle. The recent upward trend in production (estimated 500 t/a worldwide) [15] and application resulted in an increasing release of silver nanoparticles (AgNP) as well as of ionic silver into the environment as can be seen from elevated levels of silver in the aquatic environment [16–19]. Continuous input of silver can cause severe changes in species composition and species succession in phytoplankton communities [9, 20]. Ionic silver thus has the potential to disrupt their internal dynamics and feedbacks, to upset the microbial loop and to undermine the entire plankton community on which aquatic food webs are based. Silver has been shown to affect particularly metabolic pathways associated with photosynthesis and with that specifically phytoplankton [9, 21]. A binding of silver ions to chlorophyll and a decreasing efficiency of photosynthesis with increasing silver exposure might be causative [9].

The chrysophyte *Poterioochromonas malhamensis* is a widely used model organism in microbial ecology. *P. malhamensis* can utilise a wide selection of different organic food sources and can be considered a food source generalist [8]. Different *P. malhamensis* isolates have been shown to assimilate the main part of their required carbon via phagotrophy or osmotrophy and only to a minor extent by photo—autotrophy [8, 22–27]. *P. malhamensis* does not grow but sustains cell metabolism with the help of light-derived energy when organic carbon sources are limited [28]. When grown osmotrophically, i.e. in the absence of particulate food, the growth of the mixotrophic *Poterioochromonas malhamensis* is limited by the organic matter [28]. In general, data so far indicate that with increasing importance of phototrophic nutrition (*Spumella* / *Paraphysomonas* → *Poterioochromonas* → *Ochromonas* → *Dinobryon*) there is a parallel decrease in maximum growth rate [8]. This is expected, since the development of a photosynthetic apparatus (chloroplasts, enzymes) is considered to be much more energetically expensive than heterotrophic degradation of food (e.g. [29]). The effect of pollutants, in particular of silver, may therefore depend on the nutritional mode, i.e. differ between dark and light treatments.

In this study, we extend existing transcriptome and metatranscriptome studies on the physiological effect of silver by a controlled proteomic time course experiment. We address the effects of light and silver stress on the mixotrophic protist *Poterioochromonas malhamensis* using label-free quantitative mass spectrometry. The impact of light, near the light compensation point of *P. malhamensis*, and silver stress are assessed and discussed with regard to their metabolic implications. Since light is not essential for growth in *P. malhamensis* with enough organic carbon sources available, we hypothesize a possible damage to the photosystem due to silver should have a low impact on the primary metabolism. We firstly focus on the effect of light in general and secondly analyse the effect of silver with respect to these findings. Additionally, we discuss the potential of a proteomics approach for such an analysis.

## Materials and Methods

### Cultivation and sample preparation

The flagellate *Poterioochromonas malhamensis* strain DS originates from the mesotrophic Lake Constance, Germany. In Essen, *P. malhamensis* is permanently cultivated as an axenic culture in organic NSY medium [30]. This strain has been used in a variety of laboratory studies under the name *Ochromonas* sp. (e.g. [26, 31]) and under the name *Ochromonas sp.* strain DS [30, 32, 33]. All samples were taken under sterile conditions. Depending on cell density (300,000 to 1,000,000 cells/ml), we took 50 to 150 ml of culture to receive an equally large cell pellet for proteome analysis (centrifuged at 4,400 rcf, Rotina 35 R, Hettich Lab Technology). Pellets were washed three times in 75% ethanol ahead of further analyses.

### Experimental setup

**Fig 1:**
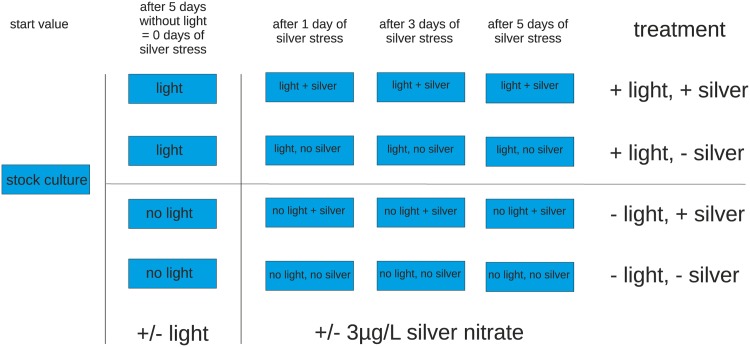
Experimental setup of light and silver nitrate exposure of cultures. The experiment was carried out in four different treatments, each treatment in four replicates (i.e. each blue box represents four replicates). First, light/no light was applied for 5 days. Then silver nitrate was added to two of the four treatments and samples were taken after 1, 3 and 5 days.

All *P. malhamensis* treatments were kept at 18°C and 35 µE m^-2^ s^-1^ light intensity in a 16/8h light-dark-cycle. For the experiments we chose a light intensity approximately corresponding to light compensation point, i.e. 35 µE m^-2^ s^-1^. The stock culture was incubated until reaching a cell density of 300,000 cells per ml (start value, SV), which corresponds to the early stationary phase. Thereupon, the stock culture was split up in 16 subcultures in 750 ml culture bottles. All cultures were kept at the same temperature for five days, but 8 cultures were kept in darkness and the other 8 in light. After five days samples were taken from the cultures for mass spectrometry (MS) measurements. This marked day 0, after which silver was added as a second stressor. To half of the cultures in dark and half of the cultures in light silver nitrate was given at an end concentration of 3 µg/l. Silver concentrations of 5 µg/l and below have been shown to affect both phytoplankton community composition and community gene expression profiles [9]. We thus decided for 3 µg/l Ag^+^ in order to induce a sub-lethal response to silver stress. Samples for MS measurement were taken 1, 3 and 5 days after silver application. This resulted in one start value measurement, in 8 replicates for light and dark at day 0 and for the time points 1, 3 and 5 in four replicates for the conditions light and silver (LS), light but no silver (LN), darkness with silver (DS) and darkness and no silver (DN) (see Fig 1). Replicate IDs consist of treatment (LS, LN, DS, DN) + replicate (A, B, C, D) + sampling day (0, 1, 3, 5). If, in the following, all replicates of a treatment are considered, the letter for the replicate (A-D) will be skipped.

### Mass spectrometry

#### Cultivation and sample preparation

The frozen cell pellets were warmed to room temperature and taken up in 1 ml phosphate buffered saline (PBS). In order to break the cells, release the proteome and to shear the genomic DNA, the samples were lysed by ultrasonification in a Bioruptor® (Diagenode) for 5 min. The turbid solution was cleared by centrifugation (12,000 rcf, 4°C, 5 min). The supernatant was transferred to a fresh 1.5 ml Eppendorf tube. The protein concentration was determined by using a modified Bradford assay (RotiNanoquant; Roth) following the manufacturer’s instructions.

#### Reduction/alkylation and tryptic digestion

We first removed an aliquot corresponding to 50 *μ*g total protein content from each sample and performed an acetone precipitation (ratio of sample to acetone = 1:4). The samples were vigorously stirred and stored over night at -20°C to completely precipitate all proteins. After centrifugation (18,000 rcf, 4°C, 5 min) the supernatant was aspirated and the pellet dried at room temperature for 10 min. The pellets were taken up in 25 *μ*L 6 M urea in 100 mM ammonium bicarbonate (ABC) and extensively vortexed. The solutions were cleared by centrifugation and the supernatants were transferred to 96-well-plates (Eppendorf). The proteins were reduced by addition of dithiothreitol (DTT, 8 mM final) and incubation at 37°C for 30 min and secondly alkylated by addition of iodoacetamide (IAM, 16 mM final) and incubation at 30°C for 30 min. In order to quench the excess IAM, we added more DTT to a final concentration of 24 mM. After this treatment 500 ng Lys-C (1:100; Wako Laboratory Chemicals) was added and the samples were incubated for 3 h at 37°C. The samples were diluted to 25 mM ABC and 1.5 M urea. 800 ng sequencing grade Trypsin (1/60; Promega) were added and the samples were incubated over night at 37°C while shaking. On the next morning, the samples were acidified by adding formic acid solution (FA, final 0.5% v/v).

#### Sample clean-up for liquid chromatography and mass spectrometry (LC-MS)

Acidified tryptic digests were desalted on home-made C18 StageTips as described [34]. On each 2 disc StageTip we loaded around 15 *μ*g peptides (based on the initial protein concentration). After elution from the StageTips, samples were dried using a vacuum concentrator (Eppendorf) and the peptides were taken up in 10 *μ*L 0.1% formic acid solution. LC-MS/MS experiments were performed on an Orbitrap Elite instrument (Thermo; [35]) that was coupled to an EASY-nLC 1000 liquid chromatography (LC) system (Thermo). The LC was operated in the one-column mode. The analytical column was a fused silica capillary (75 *μ*m × 38 cm) with an integrated PicoFrit emitter (New Objective) packed in-house with Reprosil-Pur 120 C18-AQ 1.9 *μ*m resin (Dr. Maisch). The analytical column was encased by a column oven (Sonation) and attached to a nanospray flex ion source (Thermo). The column oven temperature was adjusted to 45°C during data acquisition and in all other modi at 30°C. The LC was equipped with two mobile phases: solvent A (0.1% formic acid, FA, in water) and solvent B (0.1% FA in acetonitrile, ACN). All solvents were of Ultra Performance Liquid Chromatography (UPLC) grade (Sigma). Peptides were directly loaded onto the analytical column with a maximum flow rate that would not exceed the set pressure limit of 980 bar (usually around 0.3 − 0.6 *μ*L/min). Peptides were subsequently separated on the analytical column by running a 240 min gradient of solvent A and solvent B (start with 7% B; gradient 7% to 35% B for 220 min; gradient 35% to 100% B for 10 min and 100% B for 10 min) at a flow rate of 300 nl/min. The mass spectrometer was operated using Xcalibur software (v2.2 SP1.48). The mass spectrometer was set in the positive ion mode. Precursor ion scanning was performed in the Orbitrap analyzer (FTMS) in the scan range of m/z 300-1,800 and at a resolution of 60,000 with the internal lock mass option turned on (lock mass was 445.120025 m/z, polysiloxane) [36]. Product ion spectra were recorded in a data dependent fashion in the ion trap (ITMS) in a variable scan range and at a rapid scan rate. The ionization potential (spray voltage) was set to 1.8 kV. Peptides were analysed using a repeating cycle consisting of a full precursor ion scan (1.0 × 10^6^ ions or 30 ms) followed by 15 product ion scans (1.0 × 10^4^ ions or 50 ms) where peptides are isolated based on their intensity in the full survey scan (threshold of 500 counts) for tandem mass spectrum (MS2) generation that permits peptide sequencing and identification. Collision-induced dissociation (CID) energy was set to 35% for the generation of MS2 spectra. During MS2 data acquisition dynamic ion exclusion was set to 60 s with a maximum list of excluded ions consisting of 500 members and a repeat count of one. Ion injection time prediction, preview mode for the FTMS, monoisotopic precursor selection and charge state screening were enabled. Only charge states higher than 1 were considered for fragmentation.

#### Peptide and protein identification using MaxQuant

RAW spectra were submitted to an Andromeda [37] search in MaxQuant (v1.5.0.25) using the default settings [38]. Label-free quantification and match-between-runs was activated [39]. MS/MS spectra data were searched against the in-house generated peptide database of predicted candidate open reading frames from the assembled transcriptome of *P. malhamensis* (39,357 entries; NCBI BioSample ID: SAMEA3936180). All searches included a contaminants database (as implemented in MaxQuant, 267 sequences). The contaminants database contains known MS contaminants and was included to estimate the level of contamination. Andromeda searches allowed oxidation of methionine residues (16 Da) and acetylation of protein N-terminus (42 Da) as dynamic modification and the static modification of cysteine (57 Da, alkylation with iodoacetamide). Enzyme specificity was set to “Trypsin/P”. The instrument type in Andromeda searches was set to Orbitrap and the precursor mass tolerance was set to ±20 ppm (first search) and ±4.5 ppm (main search). The MS/MS match tolerance was set to ±0.5 Da. The peptide spectrum match false discovery rate (FDR) and the protein FDR were set to 0.01 (based on target-decoy approach). Minimum peptide length was 7 amino acids. Peptides were assembled into proteins and proteins sharing the same identified peptides were grouped together into one protein group. For label-free protein quantification (LFQ) unique and razor peptides were allowed. Modified peptides were allowed for quantification with a minimum score for modified peptides of 40. For convenience we will talk of proteins instead of protein groups hereafter, but it has to be noted that these might comprise several proteins with shared peptides.

The mass spectrometry proteomics data have been deposited to the ProteomeXchange Consortium via the PRIDE [78] partner repository (https://www.ebi.ac.uk/pride/archive/) with the dataset identifier PXD005146.

### Analysis of identified proteins

The assembled and annotated transcriptome of *P. malhamensis* was used to assign gene names, gene symbols, orthology and pathway information from the Kyoto Encyclopedia of Genes and Genomes (KEGG release 2014-06-23, [40]) to the identified proteins. For the KEGG annotation an E-value threshold of 10^−5^ was used. The analyses were performed only for proteins with annotated transcript matches in the reference transcriptome. For pathway analyses the KEGG Orthology information was used, which assigns cross-species orthology information to genes by a KEGG Orthology identifier (KO ID). These KO IDs allow the mapping of the proteins via the transcript annotation to KEGG reference pathways. The KEGG pathway analyses were restricted to the categories Metabolism, Genetic Information Processing, Environmental Information Processing, Cellular Processes and Organismal System. All analyses were performed and plots were generated with the R statistical framework (v3.2.4, [41]) and ggplot2 (v2.1.0, [42]).

#### Presence-absence analysis

A presence-absence analysis of proteins was performed to describe the proteomes and compare the coverage of KEGG pathways with those of the reference transcriptome. For pathway annotation the KEGG BRITE functional hierarchy system level A and B were used. Furthermore, this data was used to detect whether silver and light treatment switches the protein synthesis of specific proteins on or off. For protein presence-absence analysis proteins which could be identified with a minimum of two unique peptides were used. For each KEGG pathway the proteins identified by MS were counted. Statistical tests of differences between treatments were carried out on total KO ID occurrence counts and at pathway level using the generalized linear model (GLM) test of the R package edgeR (v3.12.0, [43]).

#### Differential expression analysis

The LFQ intensities from the quantitative MS were used to perform expression analyses. Replicate 4 of the treatment dark and no silver at time point 3 (sample DND3) was removed due to a high deviation from the intensity values of the other replicates (see sample 48 (bottom) in S1 Fig). For the other samples raw intensities were filtered to exclude hits for transcripts with missing values in more than one replicate and remaining missing values were imputed using k-nearest neighbour imputation from the R package impute (v1.42.0, [44]) after normalization. The raw intensities were normalized by applying a variance stabilization method implemented in the R package limma (v3.26.9, [45, 46]). Limma was also used to perform statistical testing of differential protein expression. For principal component analysis (PCA) and redundancy analysis (RDA), the R package vegan (v2.3-2, [47]) was used. By the KO identifiers the significant differentially expressed proteins were assigned to KEGG reference pathways and an in-house R package was used to colour differentially expressed proteins in the KEGG pathway maps and conduct a pathway enrichment analysis using a hypergeometric test. To visualize the pathways for publication, we used Cytoscape (v3.3.0, [48]) and the Cytoscape app KEGGscape (v0.7.0, [49]).

## Results

During the whole experiment, the strain *P. malhamensis* DS showed a more agitated movement and higher cell numbers in the light treatments. From a starting value of about 300,000 cells per ml, cell numbers in the treatments in light increased and reached a constant number of about 1,000,000 cells per ml (irrespective of silver application). The cell numbers of the dark treatments stayed at a value of about 300,000 cells per ml and even further decreased under silver application to a value of 200,000 cells per ml. Thus, in the dark treatments in contrast to the light treatments a difference in cell numbers with and without silver was visible. To understand these effects on the molecular level proteins were analysed and quantified with mass spectrometry, of which the results are described in the following.

### Presence-absence analysis

**Fig 2:**
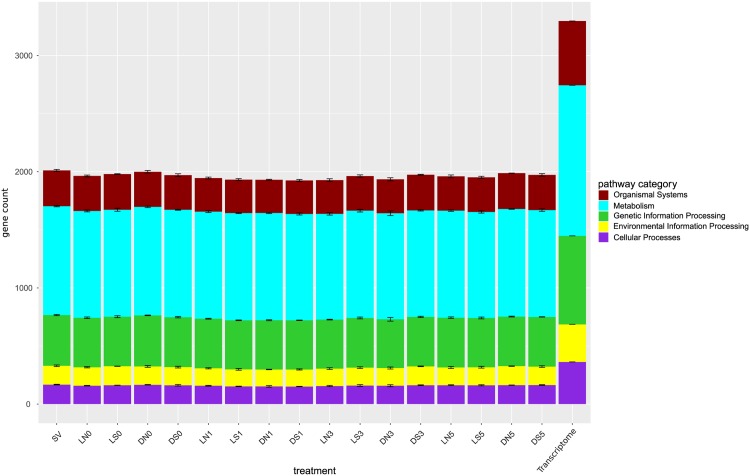
Assignment of protein matches to KEGG pathways A. Assignment of uniquely counted genes for each treatment to the KEGG BRITE functional hierarchy level A: Metabolism, Genetic Information Processing, Environmental Information Processing, Cellular Processes and Organismal System. The median over the replicates for each treatment is shown with standard deviation. Treatments are labelled according to the following scheme: light and silver (LS), light but no silver (LN), samples in darkness with silver (DS) and in darkness and no silver (DN). Treatment IDs consist of treatment (LS, LN, DS, DN) + sampling day (0, 1, 3, 5). At day 0 no silver was added yet, therefore LN0 = LS0 and DN0 = DS0, but they were already split up in 4 replicates each. SV represents the start value and Poterioochromonas DS the counts per pathway category for the transcriptome.

Altogether, we could identify 3,952 protein matches by mass spectrometry using the assembled transcriptome of *P. malhamensis* as reference, which comprises in total 39,537 transcripts. The number of detected proteins is comparable or higher to other proteomic studies that use transcriptomes or genomes for the identification, yielding between 660 to 3,886 proteins [50–54]. Considering the different treatments, the number of identified proteins ranges on average between 3,582 proteins within treatment DN3 to 3,837 proteins within treatment SV. The filtered *P. malhamensis* transcriptome contains 14,726 transcripts with a KEGG annotation E-value ≤10^−5^. We excluded all protein matches with a KEGG annotation E-value >10^−5^, yielding from 4,128 KO IDs within treatment LN3 to 4,486 KO IDs within treatment SV. In total 925 KO IDs within treatment LN3 to 962 KO IDs within treatment SV were assigned to one or more pathways, resulting in total pathway matches from on average 1,924 within treatment DS1 to 2,011 within treatment SV (Fig 2). At the level of KEGG BRITE functional hierarchy system A, on average 47% of the KO IDs were found in the category Metabolism followed by the categories Genetic Information Processing with 22%, Organismal System with 15% as well as Cellular Processes and Environmental Information Processing with 8% assignments. In comparison, the reference transcriptome had 1,599 unique KO IDs with 3,297 matches to the five KEGG categories: Metabolism (39%), Genetic Information Processing (23%), Organismal System (17%), Cellular Processes (11%) and Environmental Information Processing (10%), see Fig 2. Thus, the proteome covers a considerable amount of the transcriptome with 59% of all identified KO IDs. Using mass spectrometry, Bai et al. [53] identified about 40% of the genes in *Aspergillus flavus*, while [52] show for the sperm cells of *Caenorhabditis elegans* that 1080 of 2511 (approx. 30%) protein annotations overlap with the transcriptome. A review on Apicomplexa proteome and transcriptome studies, estimates the proportion of the covered proteome in 6 studies to lie between 10 to 45% [54].

**Fig 3:**
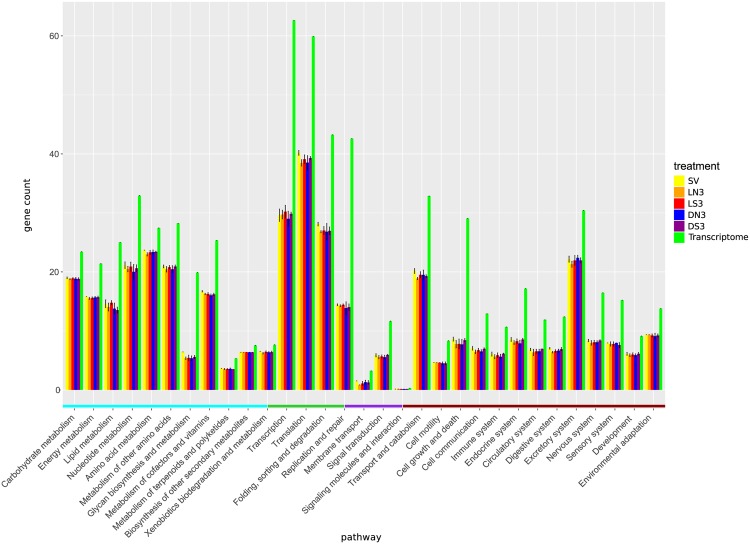
Assignment of protein matches to KEGG pathways B. Assignment of uniquely counted genes for time point 3 to KEGG pathway groups of hierarchy level B. Colours of pathways on x-axis indicate KEGG hierarchy level A from Fig 2. The median over the replicates for each treatment is shown with standard deviation. Treatments are labelled according to the following scheme: light and silver (LS), light but no silver (LN), samples in darkness with silver (DS) and in darkness and no silver (DN). Treatment IDs consist of treatment (LS, LN, DS, DN) + sampling day (3). SV represents the start value and *P. malhamensis* DS the counts per pathway category for the transcriptome.

Furthermore, we assigned uniquely counted KO IDs of the reference transcriptome and all treatments to 298 KEGG pathways (KEGG BRITE functional hierarchy level C) which were summarized to 31 pathway groups (KEGG BRITE functional hierarchy level B) within the five KEGG categories (KEGG BRITE functional hierarchy level A) to calculate the coverage of each pathway. On pathway group level the treatments of different sampling days had very similar coverages. Therefore, we exemplarily illustrated the treatments of day 3 (Fig 3). For the pathway groups of the KEGG database, we found diverging coverages from those of the reference transcriptome. Many pathway groups showed an approximately 5–10% lower coverage than the corresponding ones of the reference transcriptome. Especially pathways included in the KEGG category Genetic Information Processing i.e. transcription and replication and repair as well as pathways of the group “Cell growth and death” of the KEGG category Cellular Processes had an approximately 25% lower coverage than the reference transcriptome. In contrast, the pathway groups “Metabolism of terpenoids and polyketides”, “Biosynthesis of other secondary metabolism” and “Xentobiotics biodegradation and metabolism” of the KEGG category Metabolism as well as the pathway group “Membrane transport” of the KEGG category Environmental Information Processing showed only a 2% decrease in coverage. In addition to the pathways of the previously named pathway groups we identified on pathway level similarly covered pathways like glycolysis, citrate cycle, biosynthesis and degradation of fatty acids, many amino acids and ketone bodies as well as photosynthesis, translation and regulatory systems like the proteasome.

The statistical tests for each time point with a generalized linear model that tested for differences between all treatment combinations did not yield significant results, neither based on total gene counts nor pathway coverage.

### Expression analysis

**Fig 4:**
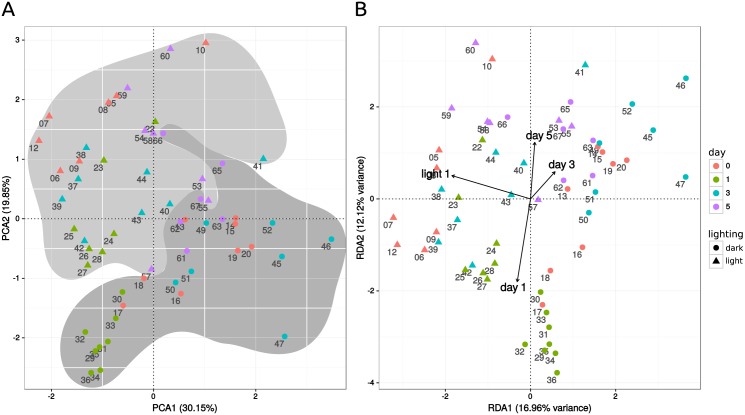
Ordination plots. (A) Principal component analysis (PCA) and (B) redundancy analysis (RDA) of normalized intensity matrix. Samples are depicted as numbers, different days are indicated by colours and symbols show samples kept under dark (circle) and light (triangle) conditions. The coloured background indicates the separation between samples in light (light grey) and dark (dark grey) conditions in the PCA. Arrows in (B) show significant factors of the reduced model. Samples were numbered for readability according to S1 Table.

Raw LFQ intensities were measured with quantitative mass spectrometry for 63 samples and proteins mapping to 4,829 transcripts. After filtering of missing values, imputation and normalization (see Methods) 2,297 proteins remained, which were subsequently analysed for differential protein expression. A principal component analysis (PCA) was used to visualize the amount of variance in the normalized LFQ intensities and the grouping of the samples due to differing intensities. The strongest effect can be seen on the first two axes of the PCA, which pronouncedly shows the difference between the light and dark treatments (Fig 4A). Furthermore, a redundancy analysis (RDA) including, supplementary to the LFQ intensities, the factors day (1, 3, 5), light conditions (L, D) and silver treatment (S, N) was applied. A stepwise model reduction of the complete initial model resulted in an overall model with day and light conditions as significant factors (p-values ≤ 0.005, see Fig 4B).

The significant factors can be assessed using the RDA, but it is unclear which proteins are responsible for the differences. This can be analysed by testing for significant differences in protein intensities between the treatments. To assess differential expression a linear model was used and the effects listed in Table 1 were tested for each time point. The strongest effect was visible on day 3. All tested contrasts showed the highest number of significant differentially expressed proteins (adj. p-value <0.01) at this time point.

**Table 1:** Number of differentially expressed proteins at all time points.

Effect Nr.	Effect	Explanation	Day 1	Day 3	Day 5
I	LN vs. DN	Light effect, no silver	90	720	0
II	LS vs. DS	Light effect, silver	61	114	32
III	LS vs. LN	Silver effect, light	0	0	8
IV	DS vs. DN	Silver effect, dark	0	104	0
V	(LS vs. LN) vs. (DS vs. DN)	Difference of silver effect in light vs. dark	0	187	0

Although light caused the severest changes in expression, at time point 3 the effect of silver for the dark samples and the difference of the silver effect in light and dark was strong as well. A beta-uniform mixture model (R package BioNet v1.29.1, [55]) fitted to the p-value distributions showed a strong deviation from the uniform-distribution (random noise) and a high signal component for the effects I, IV and V (data not shown). The pronounced differences of the silver treatment under light and dark conditions (V) show that the organism reacts in a distinct way, possibly due to an interference of silver with enzymes necessary for the dark or light reactions of photosynthesis.

**Fig 5:**
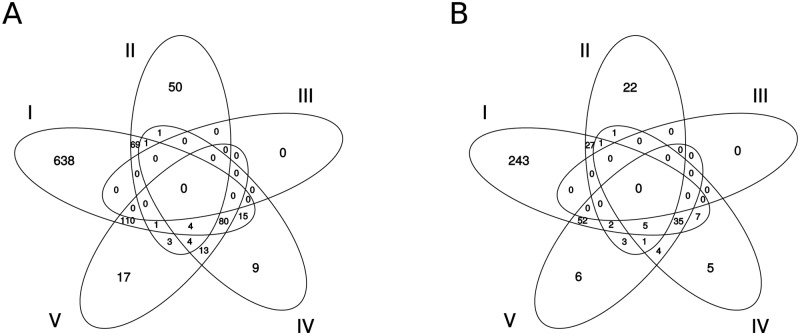
Differentially expressed KOs. Number of significantly differentially expressed proteins (adjusted p-value <0.01) annotated to (A) assembled transcripts and to (B) KEGG Orthology identifiers per tested effect at time point 3. I-V represent tested effects according to Table 1.

A large proportion of proteins was impaired by several treatments (see Fig 5). E.g. 48 KO IDs annotated to affected proteins overlapped between the light effect without silver and the silver effect in darkness for an adjusted p-value cut-off of 0.01. We also found a significant correlation (p-value <0.001) in log fold-changes (logFC) for the top 100 most differentially expressed proteins for these two treatments. However, the log fold-changes of the light effect without silver were considerably higher.

All significantly differentially expressed proteins per test at time point 3 are provided in S2 Table.

### Pathway enrichment analysis

**Fig 6:**
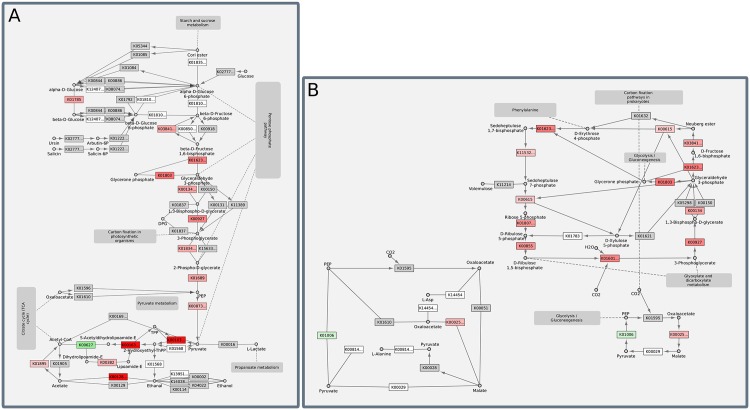
Enriched pathways in light vs. dark without silver (time point 3). Depicted are two KEGG metabolic pathways with a high enrichment in differentially expressed proteins. (A) shows the glycolysis/glyconeogenesis and (B) carbon fixation in photosynthetic organisms. Significantly differentially expressed proteins are coloured in red for a positive logFC and green for a negative logFC. Proteins in white were identified with quantitative MS, but did not show differential expression.

To identify significantly altered pathways, e.g. pathways that contain a large number of the significantly differentially expressed proteins for the light effect without silver (effect I), a pathway enrichment analysis was performed and enriched pathways were visually inspected (see Fig 6 for the two most affected pathways). The enrichment analysis on KEGG metabolic pathways yield an increased expression of proteins involved in photosynthesis, carbon fixation in photosynthetic organisms, glycolysis/gluconeogenesis, N-glycan biosynthesis, inositol phosphate metabolism, riboflavin and methane metabolism for the effect I (light). A decreased expression of several proteins was present in parts of the tricarboxylic acid (TCA) cycle and oxidative phosphorylation. Other pathways without a clear directional change in expression of a majority of significant proteins include: ascorbate and aldarate metabolism, purine metabolism, glutathione metabolism, glycerophospolipid metabolism, glyoxylate and dicarboxylate metabolism, lysosome and synaptic vesicle cycle.

In comparison, significantly differentially expressed proteins for the silver effect in darkness (IV) and also the interaction effect of silver and light (V) led to an increase in glycolysis/gluconeogenesis, parts of the TCA cycle, pyruvate metabolism, carbon fixation in photosynthetic organism and a decrease in oxidative phosporylation. Apparently, the silver treatment partially affects similar processes as the light vs. dark treatment, we will review these findings in more detail in the discussion.

For a complete list of all significantly enriched pathways per test at time point 3 see S3 Table.

## Discussion

### Potential of proteomics in microalgal ecophysiology

Quantitative protein mass spectrometry has evolved into an indispensable and versatile examination method in molecular biology research. In environmental biology including freshwater ecosystem research, its regular application is, however, very limited, mainly due to a lack of established quantitative proteomics methodologies and reference databases. In the last years, mainly (meta)genomic and/or (meta)transcriptomic approaches were used to determine or monitor overall functional capacities or nutrient/multiple stressor-induced changes in freshwater microorganisms [9, 56–59]. Transcription levels reflect the regulation of genes but the effect on the proteome and thus on the functional level remains uncertain. In fact in most instances, changes in the protein repertoire of microorganisms in a community are indirectly inferred from transcriptomics experiments thus ignoring regulation on a post-transcriptional level. In a recent study, Xie et al. [60] used in a similar approach the isobaric tags for relative and absolute quantitation (iTRAQ) technique to investigate the cellular response of the algae *Phaeodactylum tricornutum* to sub-lethal concentrations of aluminium. They found aluminium-induced toxic effects on photosynthesis leading to an increase in reactive oxygen species (ROS) and energy production. In contrast to our study, the sequenced and annotated genome of *P. tricornutum* was readily available to analyse the proteome data. Here we demonstrate that proteomics is a promising tool for ecophysiological research on microalgae if a transcriptome or genome is sequenced which can be used to construct a reference database. In our study, only 23 proteins (e.g. ATP synthase, ATPase, HSP70, actin) could be assigned to NCBI sequences in comparison to 3,952 hits on the sequenced transcriptome of *P. malhamensis*. This shows the up to now insufficiency of available database references when working with lesser studied organisms and the necessity to combine proteomic with transcriptomic or genomic data. We used assembled transcript sequences to analyse the proteomes and assign functional information on genes and pathways to the measured peptides. Ten percent of the whole transcriptome were covered by protein hits. Since alternative splicing leads to different transcript variants of one gene and thus proteins, summarizing the protein hits on gene-level (Trinity assembly components) led to a coverage of 20 percent. On the level of annotated genes with KEGG Orthology IDs the coverage even increased to about 60 percent. This shows, particularly for functional analyses, quantitative protein mass spectrometry captures a considerable amount of information.

The proteome analysis showed a similar pattern to the transcriptome analysis in the coverage of genes and pathways. In particular, the coverage of pathways participating in the basic metabolism (KEGG category Metabolism) was comparable, in contrast to pathways that are involved in Cellular Processes and Genetic Information Processing that had decreased coverages in the proteome samples. The strongest differences were found in pathways belonging to Genetic Information Processing. Possibly RNA sequencing is superior in detecting genes involved in transcriptional processes and regulation on the transcriptional level. The presence-absence analysis is an appropriate tool to describe the proteome and provide information regarding the coverage of metabolic pathways, but it does not have the resolution to detect differences in treatments. A quantitative analysis, therefore, is indispensable for a detailed comparison and assessment of expression changes under different conditions. While the presence-absence analysis did not reveal differences in protein occurrence, the LFQ intensities showed significant differences between the treatments on protein and pathway level.

### Physiological effects of environmental conditions

#### The effect of light

Our experiments demonstrate that environmental conditions have a strong quantitative effect on the proteome of the investigated microalga *P. malhamensis*. The light versus dark treatment showed pronounced differences at time point 3 with 720 proteins being significantly differentially expressed. Specifically, pathways of the energy and primary carbon metabolism were differentially affected by light (see S3 Table). This is in accordance with studies on light-activated heterotrophic cyanobacteria, indicating a shift in the central carbon metabolism in response to trophic change [61, 62]. It is accompanied by an enhanced glycolysis, oxidative pentose phosphate pathway as well as tricarboxylic acid cycle during heterotrophic growth. A recent study on mixotrophic chrysophytes with a primarily phototrophic mode of nutrition also shows alterations in the expression level of genes involved in carbon utilization pathways under light and dark conditions [63]. Although, *P. malhamensis* does not grow but sustains cell metabolism with the help of light-derived energy when organic carbon sources are limited [28], the high amount of differentially expressed proteins suggest a heavy usage of the photosynthesis apparatus to generate energy and storage molecules. Particularly, photosynthesis and calvin cycle are increased in light to generate energy via photosynthesis for primary production. If photosynthesis is the main carbon source, glycerate-3-phosphate will be produced as an intermediate to enter glycolysis/gluconeogenesis [63]. The energy surplus allows the storage of glucose by gluconeogenesis. It is more likely that gluconeogenesis is affected instead of glycolysis due to an increased expression of the fructose-bisphosphatase, driving the reactions towards the storage of glucose potentially in the form of chrysolaminarin, a storage polysaccharide typically found in photosynthetic chrysophytes [64]. In addition, the decreased protein degradation and two key enzymes of the glyoxylate cycle, link beta-oxidation, the degradation of fatty acids, with gluconeogenesis and carbon storage. It has been found by Li et al. [65] that biosynthesis of starch and lipids benefits in the microalga *Chlorella sorokiniana* from a more active photosynthesis. Although, the increased expression of enzymes of the glycerophospholipid metabolism indicates a build-up of fatty acids for membrane biosynthesis potentially for growth and cell division, no other pathway indicates growth such as the biosynthesis of amino acids, nucleotides or ribosomes. Proteins in the purine metabolism are differentially expressed, but only reactions related to ATP production are increased in light, while the production of adenine and guanine is decreased as well as the pentose phosphate metabolism that produces ribose 5-phosphate to form phosphoribosyl pyrophosphate with ATP for purine generation. During phototrophic growth sulfate assimilation, an energy expensive reaction, is active to produce cysteine and methionine [66]. Glutathione on the other hand can store reduced sulphur as a major reservoir from the uptake of amino acids from ingested food under the heterotrophic mode of nutrition. For the dark treatment the glutathione metabolism is enriched in differentially expressed proteins. Further, the V-type proton ATPase subunit, showing a higher expression in the dark, participates in the acidification of vesicles including lysosomes for the digestion of macromolecules and prey. Under light conditions, the increased expression of the riboflavin metabolism is necessary for chlorophyll synthesis. The riboflavin metabolism, which utilizes expensive reactions, as well as other energy expensive reactions hint to a surplus of energy and “prosperity” of *P. malhamensis* under light conditions.

#### The effect of silver

The effects of silver were less explicit. They were observable particularly in the dark treatments where the stronger light effect could not mask possible effects of silver. Here still many proteins involved in different pathways, e.g. oxidative phosphorylation, cysteine and methionine metabolism and photosynthesis-related pathways, were significantly differently expressed (see S3 Table, effect IV and V). Interestingly several proteins with thiol-groups were affected, e.g. methionine-dependent methyltransferase, glutathione reductase, glutathione S-transferase family protein protein disulfide isomerase, NADH dehydrogenase and glyceraldehyde-3-phosphate dehydrogenase (see S2 Table, effect IV and V). This can be explained by the fact that silver was found to interfere specifically with sulphur-containing molecules inside the cell [67–71]. Silver causes mis-folding and damage of proteins by binding to thiol groups, regulates the expression of proteins in ATP- and photosynthesis and replaces Cu+ in key proteins in the latter pathways in *Chlamydomonas reinhardtii* [72]. At low concentrations silver is predominantly present inside the cytosol, while at higher concentrations it can also be found in chloroplasts, where it damages the photosystem, in mitochondria, on the plasma membrane, on cytosolic membrane structures, and in vacuoles. At high concentrations silver damages irreversibly, and photosynthesis and growth get inhibited [70]. At the applied concentrations silver did not have a severely damaging effect, but a decrease in growth was observed as well as an inference with photosynthesis pathways and proteins with thiol-groups. A decreased expression of light-harvesting proteins was found in Lhcf12, LHCA1, Lhcf14, Lhcr1 and chlorophyll binding photosystem II (PSII) CP43 apoprotein. The light-harvesting complexes are sensitive to stress [72] and their potential down-regulation as well as the adverse effect on PSII lead to a partial inhibition of the photosynthetic machinery. Under dark conditions the effect on photosynthesis is dispensable in the mixotrophic organism that can utilize a heterotrophic mode of nutrition. But the effect likely also occurs in light and is only concealed by a massively increased photosynthesis and replacement of damaged proteins. Additionally, the increased lipid production under light conditions could compensate for the damage of lipids and membranes by silver ions. The low Ag^+^ concentration might also alleviate the effect seen in light in this study. The increase of the cysteine and methionine metabolism indicates that silver additionally blocks or damages the thiol-groups here and thus the production of these compounds has to be intensified. The general increased expression of proteins in other pathways can likewise be interpreted as a stress response to the presence of silver. In *C. reinhardtii* Pillai et al. [72] found that mitochondria and chloroplast, and transport to these organelles, were specifically affected by silver due to lipid peroxidation by reactive oxygen species (ROS). We could confirm an effect on chloroplast and mitochondrial proteins, such as the mitochondrial import inner membrane translocase, chloroplast ribose-5-phosphate isomerase and chloroplast ADP, ATP carrier protein. Energy supply by oxidative phosphorylation was decreased under the influence of silver, possibly further slowing down general metabolism in the presence of a stressor in addition to no-light conditions. Furthermore, presumably the storage compound of the cells, i.e. chrysolaminarin in Chrysophyceae, is used to back the cells’ metabolism in the double stress situation.

Photosynthetic pathways were affected by the single treatments of light/darkness and silver, but specifically photosynthetic pathways were also affected by interactions between the two factors (effect V). In a way this seems sensible: as photosynthesis is a light-dependent pathway a potential differential regulation of proteins in photosynthetic pathways related to silver stress has different consequences in light and in dark.

The generalisability of our findings has to be tested in follow-up studies. *P. malhamensis* is a mixotrophic algae using heterotrophy as the dominant mode of nutrition. It was not in the focus of this study to analyse to what extent other algae, specifically taxa on the phototrophic side of the spectrum, which have been investigated elsewhere [28, 63, 73–77], would respond to the tested factors. However, we would expect a higher damage with increased dependence on photosynthesis and at an increased concentration of silver. Community studies on silver effects on metatranscriptomes indicate that photosynthesis is specifically strongly affected by silver and our results are in line with these findings [9, 70]. Although, continuous input of silver can cause severe changes in species composition in phytoplankton communities [9, 20], we have evidence from community experiments with diverse stressors, that the presence of one chrysophyte might also help another to buffer the adverse effect of a stressor (pers. comm. Christina Bock). We speculate that a mixotropic species can accumulate small amounts of toxic silver without further damage and thereby reduce the toxin concentration to help the more sensitive phototrophic organism to survive. However, the accumulation might also introduce harmful implications on higher levels of the food chain.

## Supporting Information

S1 Fig Principal component analysis including all samples. Samples are depicted as numbers, different days are indicated by colours and symbols show samples kept under dark (circle) and light (triangle) conditions. Samples were numbered for readability according to S1 Table.
(EPS)

S1 Table Sample IDS. Mapping of sample IDs to treatments.
(XLSX)

S2 Table Differentially expressed proteins. Significant differentially expressed proteins for the tested contrasts I-V.
(XLSX)

S3 Table Enriched pathways. Significantly enriched pathways for the tested contrasts I-V.
(XLSX)
